# Patient experiences of information-sharing and patient-centred care across the broad landscape of primary care practice and provision: a nationally representative survey of Australian adults

**DOI:** 10.1186/s12875-024-02359-8

**Published:** 2024-05-04

**Authors:** Amie Steel, Hope Foley, Kim Graham, Joanna Harnett, Jon Adams

**Affiliations:** 1 ARCCIM, School of Public Health, Faculty of Health, University of Technology Sydney, 235-253 Jones St, Ultimo, NSW Australia; 2 School of Pharmacy, Faculty of Medicine and Health, The University of Sydney, Sydney, Australia

**Keywords:** Primary care, Health promotion, Patient education, Knowledge mobilisation, Shared decision-making, Patient expert

## Abstract

**Background:**

Australian government strategies and frameworks have been developed in recent years to encourage the integration and coordination of primary care delivery; including patient-centred approaches to clinical and preventative care, and health promotion. This study aims to explore patient experiences of information-sharing and patient-centred care across various primary care clinical settings, with a particular focus on clinical encounters with GPs, naturopaths, osteopaths and acupuncturists.

**Methods:**

Data about healthcare utilisation and experiences from a 63-item cross-sectional survey obtained from a nationally representative sample of Australian adults aged ≥ 18 years were analysed. Chi-square and Kruskal-Wallis H tests were used to explore differences in the experiences of knowledge and information sharing during GP consultations among those who also consulted with a naturopath, osteopath or acupuncturist, compared those who had not. Logistic regression was used to investigate correlations between participants perceptions about GP consultation outcomes, and the GP’s information-sharing behaviour or perceived experience of patient-centredness.

**Results:**

Across 2354 participants, verbal explanation (76.3%) and/or individualised handouts (16.8%) were the most common type of information shared in GP consultations. Individuals who consulted with a GP and a naturopath, an osteopath, or an acupuncturist reported a lower rate of receiving a verbal explanation from their GP but higher rate of receiving other types of information sources including handouts. Over one quarter of study participants who visited a GP did not discuss any of their health information with their GP. Information sharing was lower for individuals who also visited a naturopath, osteopath or acupuncturist. Participants scored their consultations with a GP as patient-centred, but these scores were lower among participants who also consulted with at least one other primary care practitioner type included in the study.

**Conclusions:**

Public health and health services researchers, policymakers and leaders of primary care professions have a role and responsibility to ensure practitioners are confident and competent in sharing health information with their patients that considers their health literacy needs, and the importance of patient-centred care. Research focussed on a more in-depth understanding of the differences and relationships observed across the primary care landscape in this study is recommended.

**Supplementary Information:**

The online version contains supplementary material available at 10.1186/s12875-024-02359-8.

## Introduction

Primary care – the health care that people visit first in their community [1] - is the foundation of universal health care efforts in Australia [2] and represents frontline health care services [3]. General practice is integral to these frontline services, acting as both an initial point of contact with the health system and a central point of referral to other health services [3]. Alongside general practice, other important primary care services are provided by pharmacists, allied and complementary health providers, nursing practitioners, specialists, and community and Indigenous health workers [3]. While the Australian health system typically delivers high quality primary care, challenges arise from the impacts of an ageing population, the shifting burden and complexity of disease, policy and funding changes, and increasing demand for primary care services [4]. In response to these challenges, practice and policy often focuses on encouraging the integration and coordination of health care delivery, patient-centred approaches to clinical care, and the importance of health promotion and preventive care [2, 5, 6] .

The Australian Federal government have identified preventive health and health promotion initiatives as crucial to successfully address the multifaceted challenges surrounding contemporary health by aiming to reduce disease burden, health service demand and health inequity [5, 6]. The success of such initiatives may partly rely on the development of broader shifts in self-care behaviours amongst individuals and communities – self-care denoting the ability of individuals to achieve, maintain and promote optimal health by performing relevant self-directed actions and decision-making [7]. Self-care practices support the effectiveness of primary care by enhancing the uptake of health promotion initiatives through active patient participation in health management [8]. However, implementing and sustaining self-care behaviours relies on an individual’s health literacy which can be defined as “how people understand information about health and health care, and how they apply that information to their lives, use it to make decisions and act on it” [9]. Health literacy is developed through information-sharing [10, 11] and shared decision-making [12] between primary care providers and patients. However, it may also be developed through knowledge mobilisation, which relates to the sharing and use of knowledge and information between different stakeholders [13], and as such encompasses information shared by patients as well as information shared with patients [13, 14].

While health literacy is an important part of health behaviour, it can be challenging to foster in the age of “Dr Google” and the “patient expert”, where individuals may access online health information sources of varied and in some cases questionable quality or accuracy [15]. In a health information landscape characterised by online sources of diverse quality, health information provided by trusted primary care practitioners is highly valued by patients [15], highlighting the importance of such providers in supporting population health literacy. Patient-centred delivery of primary care may be particularly beneficial to support the development of health literacy and subsequent health-promoting self-care behaviours [16], as the individualised approaches allow information-sharing and decision-making to be tailored to patients’ health literacy needs [17]. The delivery of patient-centred care and broader capacity to promote health literacy through information-sharing may differ across primary care settings [18]. Health professions which characterise themselves by holistic practice and tend to include greater patient-provider interaction in their consultations – such as naturopaths, osteopaths, and acupuncturists – have been shown to deliver patient-centred care and share health information with patients to a greater degree than general practitioners in Australia [18, 19]. Furthermore, international research has highlighted the priority placed on patient education among these other types of health professions [19].

In Australia, professions such as osteopathy, acupuncture and naturopathy often operate outside of conventional public health settings, yet they are nonetheless utilised at substantial rates (5.4%, 7.9% and 6.2%, respectively, within preceding 12 months) [20]. There is also evidence that these professions perform services consistent with primary care roles, such as treatment and management of both acute and chronic health conditions, health promotion support across the lifespan, health education and support with changing behaviours, and acting as a point of referral to other health professionals [20–22]. Osteopathy and acupuncture are regulated in Australia with access to private health cover, while naturopathy is not yet a registered profession, despite long-standing recommendations from relevant stakeholders, including consumer demand [23–26]. All three of these professions are commonly accessed in private ambulatory clinical settings in Australia and are associated with greater consultation length compared to general practice which may allow greater opportunity for patient-provider information sharing, patient education, and more tailored, patient-centred approaches to clinical care [18, 27]. Taken together, the practice characteristics and public utilisation of these professions represent an under-examined source of patient-centred care and information-sharing in the Australian community [19, 22].

The central role of primary care providers in health care delivery means it is important to understand how knowledge and information are currently shared and discussed in clinical consultation across different primary care settings, including but not limited to general practice. Understanding the prevailing patterns of this knowledge and information sharing has the potential to enhance patient care, safety and health outcomes. For example, it is common for individuals in Australia to use multiple forms of health care treatments or services, yet patients often fail to disclose all of their health care use to their primary care providers because they are not asked about it and are not aware this information is important [28]. While patient-centred care may be a useful approach to support more effective information-sharing and improving subsequent health outcomes [16], little research has examined whether this is occurring broadly within primary care. Research on the associations between patient-centred care, information-sharing and health literacy in primary care settings has tended to assess the impact of controlled patient-centred interventions, rather than exploring existing patient experiences [29]. Moreover, the bulk of these investigations have focused only or predominantly on general practice with little attention to other forms of primary care being accessed in the community [30]. In response, this study aims to explore patient experiences of information-sharing and patient-centred care across a broad range of primary care clinical settings.

## Methods

### Aim

This study aimed to describe the patient-reported experiences of encounters with a broad range of primary care providers in Australia, with nested focus upon patient satisfaction regarding the outcomes of the encounter and how knowledge and information is shared between providers and patients. The study also aimed to compare the patient experiences and information-sharing behaviours of those patients consulting a GP compared to those consulting with other primary care providers, namely naturopaths, acupuncturists and osteopaths.

### Study design

This study analysed data from a cross-sectional survey that collected information regarding health service use and experiences of a nationally representative sample of Australian adults. Representativeness was determined using Chi squared tests comparing the sample population to National Census data [31].

### Participants

A minimum sample size of *n* = 2000 was sought, which was deemed sufficient for the conduct of inferential sub-group analyses across primary care settings, to achieve a 95% confidence interval and 5% margin of error. This calculation was based on the proportion of the Australian adult population consulting with the least-commonly accessed of the primary care professions (osteopaths, at 5.4% of population in previous 12 months), as reported in previous studies [20, 32].

#### Eligibility criteria

Individuals were eligible to participate if they were aged 18 years and over, resided in Australia, and could complete the survey in English.

#### Recruitment

Participants were recruited via a survey panel company (Qualtrics) between 4 February and 18 February 2022, using an established database of people who have expressed interest in participating in research. Participants were required to complete informed consent forms prior to undertaking the survey. A small financial incentive ($3 - $4) was provided to participants by the survey panel company, based on survey completion time, in the form of points which can be accrued and redeemed for cash in accordance with Qualtrics’ panel membership processes.

### Survey instrument

The survey instrument included 63 questions, of which 15 may have repeated up to five times, depending on the number of responses provided to previous answers. The questionnaire was informed by a previous study [20] and developed using validated measures where appropriate (outlined below). The survey instrument was piloted with individuals known to the research team who did not have any health training. These individuals included a mix of women and men and encompassed varied levels of education and age. A copy of the full survey instrument can be viewed in Supplementary File S1.

The items analysed for this paper were selected to respond to the research aim of describing patient experiences regarding information sharing in primary care clinical settings, covering four domains: *health service use*, *demographics, health status*, and *consultation characteristics*.

#### Health service use

Health service use items captured consultations with a range of health professionals in the previous 12 months, including general practitioners, naturopaths, acupuncturists and osteopaths.

#### Demographics

Participants were asked to provide information about their age (in categories), gender, relationship status, ability to manage financially, employment status, and highest qualification. They were also invited to indicate whether they held private health insurance cover or a health care card; the latter representing access to additional government-subsidies for health services and products available for individuals on low-incomes (e.g., free or reduced-cost consultations with health care providers, diagnostic testing and imaging, and reduced-cost pharmaceutical prescriptions).

#### Health status

Participants were presented with two validated instruments to assess wellbeing and quality of life. The first is the Personal Wellbeing Index [33], which includes nine items measuring perceived satisfaction with various life and personal circumstances encompassing standard of living, health, personal relationships, personal security, and spirituality. Health-related quality of life was measured using SF-20 [34], which evaluates quality of life across six domains: physical health, role function, social function, mental health, health perception and pain.

#### Consultation characteristics and experience

Patient experiences of care were assessed using the Patient-Centred Care Scale [25]. The scale was presented to respondents for each health professional they reported visiting in the previous 12 months. Participants were also asked to indicate the reason for their visit (acute illness, chronic illness, wellbeing, or other), the consultation format (in person or telehealth) and the consultation outcome (e.g., *I was provided a formal diagnosis of my health condition*, or *I am still bothered by the same concern*) for each practitioner-type they consulted. The survey instrument also included items that asked about knowledge and information sharing through the clinical encounter including the type of information provided by the health professional (e.g., a verbal explanation, a pre-prepared handout) and the source of information the participant shared with the health professional (e.g., personal experience, books, social media).

### Statistical analysis

All survey responses (*N* = 2569) were cleaned to remove duplicate or unreliable responses. New variables were generated to categorize the chronic illness items (e.g., cardiovascular conditions, musculoskeletal conditions, respiratory conditions) and relationship status to broader groups. The Personal Wellbeing Index was scored in accordance with the instrument requirements [35] and categorised as low ( < = 50), moderate (51 to 80), or high (> 80) wellbeing. Mean scores were calculated for each domain of the SF-20 items as outlined in the instrument guide [34]. Missing data were excluded from all analysis. The demographic characteristics of participants with missing results for analysis of information-sharing and patient-centred care were identified through chi-square tests.

Descriptive analysis was undertaken for all variables of interest, as outlined in the preceding *Survey instrument* section. Categorical variables were reported as frequencies and percentages. Continuous variables were reported by means and standard deviations. Tests of association were undertaken comparing the demographic and health characteristics of individuals consulting with a GP and the rest of the population. These tests were repeated for individuals consulting with a naturopath, acupuncturist or osteopath. Chi-square tests were used to test associations between categorical variables. Kruskal-Wallis H test was used to test associations between categorical and non-parametric continuous or ordinal variables. Additional tests of association were conducted to compare the differences in consultation experience and knowledge or information sharing in GP consultations among individuals who consulted with a naturopath, osteopath or acupuncturist *and* consulted with a GP, compared to individuals who consulted with a GP without consulting the other health professional.

Logistic regression was used to investigate correlations between participants’ perceptions of their outcomes of consultations with a GP and the GP’s information-sharing behaviour or the participants’ experience of patient-centredness. Specifically, the relationship between participants’ views on whether they received an adequate explanation from their consultation with the GP was tested against the different types of information sources provided by the GP. The correlation between participants’ perception of receiving an acceptable treatment plan and patient-centred care scale items were also tested: namely, ‘the root causes of my problems are being treated by my GP’, ‘the treatment is individualised for me at each consultation’, and ‘my GP teaches me ways to relieve symptoms myself’. The regression models were adjusted for participants’ highest education level as a proxy for health literacy and for relationship status, financial stress, and employment status to control for the potential influence of missing data. The models also controlled for consultations with the other three primary care professions of interest (naturopath, osteopath, acupuncturist).

The alpha value was set at equal to or less than 0.05.

## Results

After the survey responses were cleaned, 2354 were retained for analysis. Of these 87.8% (*n* = 2185) participants reported consulting with a GP in the previous 12 months while 6.6% (*n* = 165), 6.7% (*n* = 167), and 6.9% (*n* = 171) reported consulting with a naturopath, osteopath or acupuncturist, respectively. Participants who did not complete all survey items and had missing data excluded from some of the analyses were found to vary by gender and relationship status for individuals who consulted an osteopath, and by relationship status, financial management, and employment status for participants who consulted a GP. No differences were identified for participants with missing data who consulted a naturopath or chiropractor.

### Participant characteristics

The demographics of included participants are presented in Table 1. There were statistically significant differences in participant age (*p* < 0.001) for all groups, with participants who consulted a naturopath, osteopath, or acupuncturist commonly younger (< 45 years old) than other participants and a slightly greater prevalence of consultations with a GP among participants aged 65 years and over. Participants who reported consulting a naturopath, acupuncturist or osteopath more frequently indicated that financial management was difficult some of the time or all the time (*p* < 0.001) compared with other respondents. They also reported a higher prevalence of holding a health care card than individuals not consulting these types of health professionals (*p* < 0.001).

**Table 1:** Participant demographics

Demographics	All (*n* = 2354)	Consulted with a GP (*n* = 2067)		Consulted with a naturopath (*n* = 141)		Consulted with an osteopath (*n* = 143)		Consulted with an acupuncturist (*n* = 147)	
Gender		N (%)	P			N (%)	p	N (%)	p
*Female*	1248 (53.0)	1109 (53.7)	0.02	79 (56.0)	0.5	74 (51.8)	0.2	80 (54.4)	0.2
*Male*	1081 (45.9)	940 (45.5)		59 (41.8)		65 (45.5)		63 (42.9)	
*Non-binary*	18 (0.8)	14 (0.7)		2 (1.4)		3 (2.1)		3 (2.0)	
*Prefer to self-describe*	7 (0.3)	4 (0.2)		1 (0.7)		1 (0.7)		1 (0.7)	
Age									
*18 years to 24 years*	350 (14.4)	297 (14.4)	< 0.001	35 (24.8)	< 0.001	32 (22.4)	< 0.001	33 (22.5)	< 0.001
*25 years to 34 years*	461 (19.6)	389 (18.8)		42 (29.8)		41 (28.7)		40 (27.2)	
*35 years to 44 years*	452 (19.2)	383 (18.5)		39 (27.7)		38 (26.6)		42 (28.6)	
*45 years to 54 years*	269 (11.4)	225 (10.9)		12 (8.5)		11 (7.7)		13 (8.8)	
*55 years to 64 years*	264 (11.2)	234 (11.3)		6 (4.3)		9 (6.3)		9 (6.1)	
*65 years and over*	558 (23.7)	539 (26.1)		7 (5.0)		12 (8.4)		10 (6.8)	
Relationship status									
*Never married*	731 (31.1)	581 (28.1)	< 0.001	44 (31.2)	0.4	42 (29.4)	0.5	36 (24.5)	0.004
*Married/Defacto*	1304 (55.4)	1198 (58.0)		83 (58.9)		85 (59.4)		100 (68.1)	
*Separated/divorced/widowed*	319 (13.6)	288 (13.9)		14 (9.9)		16 (11.2)		11 (7.5)	
Financial management									
*It is impossible*	103 (4.4)	83 (4.0)	0.2	17 (12.1)	< 0.001	18 (12.6)	< 0.001	17 (11.6)	< 0.001
*It is difficult all of the time*	360 (15.3)	320 (15.5)		35 (24.8)		33 (23.1)		35 (23.8)	
*It is difficult some of the time*	712 (30.3)	628 (30.4)		52 (36.9)		54 (37.8)		55 (37.4)	
*It is not too bad*	830 (35.3)	733 (35.5)		28 (19.9)		23 (16.1)		26 (17.7)	
*It is easy*	349 (14.8)	303 (14.7)		9 (6.4)		15 (10.5)		14 (9.5)	
Employment status									
*Full time work*	771 (32.8)	659 (31.9)	< 0.001	75 (53.2)	< 0.001	76 (53.2)	< 0.001	78 (53.1)	< 0.001
*Part time work*	402 (17.1)	351 (17.0)		28 (19.9)		29 (20.3)		28 (19.1)	
*Casual/temporary work*	147 (6.2)	126 (6.1)		10 (7.1)		8 (5.6)		9 (6.1)	
*Looking for work*	192 (8.2)	158 (7.6)		12 (8.5)		13 (9.1)		10 (6.8)	
*Not in the paid workforce*	842 (35.8)	773 (37.4)		16 (11.4)		17 (11.9)		22 (15.0)	
Highest qualification									
*No formal qualification*	54 (2.3)	44 (2.1)	0.2	4 (2.8)	0.2	3 (2.1)	0.02	5 (3.4)	0.01
*Year 10 or equivalent*	287 (12.2)	253 (12.2)		10 (7.1)		14 (9.8)		15 (10.2)	
*Year 12 or equivalent*	510 (21.7)	44 (21.5)		31 (22.0)		33 (23.1)		29 (19.7)	
*Trade/apprenticeship*	166 (7.1)	154 (7.5)		14 (9.9)		17 (11.9)		20 (13.6)	
*Certificate/diploma*	594 (25.2)	523 (25.3)		21 (22.0)		25 (17.5)		28 (19.1)	
*University degree*	545 (23.2)	483 (23.4)		34 (24.1)		31 (21.7)		32 (21.8)	
*Higher university degree*	198 (8.4)	166 (8.0)		17 (12.1)		20 (14.0)		18 (12.2)	
Private health insurance	1132 (48.1)	1022 (49.4)	< 0.001	91 (64.5)	< 0.001	85 (59.5)	0.005	99 (67.4)	< 0.001
Health care card	1343 (57.1)	1205 (58.3)	0.001	100 (70.9)	0.001	100 (69.9)	0.001	97 (66.0)	0.02

Table 2 presents the participants’ health status. More than three quarters of participants scored either moderate (56.3%) or high (22.2%) on the Personal Wellbeing Index. A greater proportion of participants who consulted a naturopath received a low or moderate PWI score, and a lesser proportion received a high PWI score compared with the rest of the sample (*p* = 0.04). An association between health-related quality of life was found when comparing individuals who consulted with one of the primary care professions studied, and the total sample. Individuals who consulted a naturopath, osteopath or acupuncturist scored higher for the pain domain and lower across all other domains compared with the general population (*p* < 0.001). Participants who consulted a GP scored lower in physical health and role function, and higher in health perception and pain domains (*p* < 0.001). They did not report a difference in social function or mental health.

**Table 2:** Participant health status

	All (*n* = 511)	Consulted a GP		Consulted a naturopath		Consulted an osteopath		Consulted an acupuncturist	
	*N (%)*	*N (%)*	*p*	*N (%)*	*p*	*N (%)*	*p*	*N (%)*	*p*
Personal Wellbeing Index									
*Low ( < = 50)*	540 (21.5)	446 (20.8)	0.04	38 (23.8)	0.04	40 (24.5)	0.5	38 (22.9)	0.9
*Moderate (51–80)*	1413 (56.3)	1204 (56.2)		99 (61.9)		91 (55.8)		92 (55.4)	
*High (> 80)*	558 (22.2)	492 (23.0)		23 (14.4)		32 (19.6)		36 (21.7)	
Health-related quality of life (SF-20)	*Mean (SD)*	*Mean (SD)*	*p*	*Mean (SD)*	*p*	*Mean (SD)*	*p*	*Mean (SD)*	*p*
*Physical health*	70.8 (32.1)	69.6 (32.1)	< 0.001	48.4 (29.5)	< 0.001	47.5 (27.8)	< 0.001	49.6 (29.4)	< 0.001
*Role function*	70.9 (39.3)	69.6 (39.8)	< 0.001	45.2 (34.7)	< 0.001	41.8 (34.2)	< 0.001	42.8 (34.6)	< 0.001
*Social function*	72.2 (33.1)	72.3 (32.7)	0.06	41.6 (33.8)	< 0.001	41.3 (34.5)	< 0.001	40.5 (34.4)	< 0.001
*Mental health*	59.1 (16.8)	59.0 (15.7)	0.6	46.3 (19.7)	< 0.001	46.5 (20.3)	< 0.001	48.2 (20.0)	< 0.001
*Health perception*	50.3 (11.8)	50.9 (11.4)	< 0.001	45.0 (14.4)	< 0.001	44.9 (14.8)	< 0.001	46.3 (14.6)	< 0.001
*Pain*	26.8 (20.4)	27.8 (20.3)	< 0.001	36.4 (20.6)	< 0.001	35.3 (21.6)	< 0.001	35.8 (20.6)	< 0.001

### Consultation characteristics

Consultation characteristics were reported for all primary care professions studied (see Table 3). Chronic illness was identified as the reason for the consultation for approximately half of the participants who reported consulting with each of the health professions studied (GP: 51.8%; naturopath: 55.1%; acupuncturist: 50.6%; osteopaths: 49.4%). When compared to other participants who consulted a GP, those who consulted both an osteopath and a GP reported a greater prevalence of consulting their GP for an acute illness (41.1% vs. 27.7%; *p* = 0.009). A similarly greater proportion of consultations for acute illness was found for individuals who consulted an acupuncturist and a GP compared to those consulting a GP alone (37.8% vs. 27.7%; *p* = 0.05).

**Table 3:** Consultation characteristics

Reason for visit	Consulted with a GP (*n* = 2002)	Consulted with a naturopath			Consulted with an osteopath			Consulted with an acupuncturist		
		Characteristic of naturopath consultation (*n* = 89)	Characteristic of GP consultation (*n* = 74)	P value	Characteristic of osteopath consultation (*n* = 89)	Characteristic of GP consultation (*n* = 74)	P value	Characteristic of acupuncture consultation (*n* = 83)	Characteristic of GP consultation (*n* = 74)	P value
*For an acute illness/condition, one that lasted less than one month*	554 (27.7)	22 (24.7)	25 (33.8)	0.2	31 (34.8)	30 (41.1)	0.009	25 (30.1)	28 (37.8)	0.05
*To treat a long-term health condition (one that lasted more than one month), or its symptoms*	1036 (51.8)	49 (55.1)	37 (50.0)	0.8	44 (49.4)	42 (57.5)	0.3	42 (50.6)	38 (51.4)	0.9
*To improve wellbeing*	686 (34.3)	36 (40.5)	31 (41.9)	0.2	23 (25.8)	27 (37.0)	0.6	34 (41.0)	27 (36.5)	0.7
*Other*	251 (12.5)	1 (1.1)	6 (8.1)	0.2	3 (3.4)	5 (6.9)	0.1	2 (2.4)	6 (8.1)	0.2
Consultation format										
*In person*	1853 (92.6)	75 (84.3)	61 (82.4)	0.001	77 (86.5)	63 (86.3)	0.04	73 (88.0)	61 (82.4)	0.001
*Telehealth*	540 (27.0)	19 (21.4)	26 (35.1)	0.1	14 (15.7)	27 (37.0)	0.05	13 (15.7)	30 (40.5)	0.007
Consultation outcome										
*I was provided an adequate explanation of my health complaint*	1159 (57.9)	33 (37.1)	37 (50.0)	0.2	45 (50.6)	39 (53.4)	0.4	35 (42.8)	35 (47.3)	0.06
*I was provided a formal diagnosis of my health condition*	666 (33.3)	29 (32.6)	22 (29.7)	0.5	26 (29.2)	26 (35.6)	0.7	22 (26.5)	30 (40.5)	0.2
*I was prescribed an acceptable treatment plan to manage my health complaint*	963 (48.1)	30 (33.7)	30 (40.5)	0.2	29 (32.6)	39 (53.4)	0.4	30 (36.1)	36 (48.7)	0.9
*I am still bothered by the same concern*	397 (19.8)	17 (19.1)	13 (17.6)	0.6	16 (18.0)	19 (26.0)	0.2	21 (25.3)	12 (16.2)	0.4
*I am bothered by a new health concern*	96 (4.8)	7 (7.9)	8 (10.8)	0.01	4 (4.5)	9 (12.3)	0.002	6 (7.2)	7 (9.5)	0.06
*Other*	130 (6.5)	5 (5.6)	3 (4.1)	0.4	2 (2.3)	3 (4.1)	0.4	3 (3.6)	4 (5.4)	0.7

Participants who consulted a GP most commonly reported having an in-person consultation with the GP (92.9%). The rate of in-person GP consultations was significantly lower for individuals who also consulted a naturopath (82.4%, *p* = 0.001), an osteopath (86.3%, *p* = 0.04) or an acupuncturist (82.4%, *p* = 0.001). More than half of participants reported receiving an adequate explanation from their GP (57.9%), but less than half reported being given an acceptable treatment plan (48.1%). Approximately one third of participants who consulted a naturopath reported receiving an adequate explanation from their naturopath (37.1%) while 50.6% of participants who consulted an osteopath indicated they received an adequate explanation from their osteopath and 42.8% of acupuncturists reported the same from their acupuncturist. Participants who consulted a naturopath, osteopath or acupuncturist reported the treatment plan as acceptable in approximately one third of cases (naturopath: 33.7%, osteopath: 32.6%, acupuncturist: 36.1%). Those who consulted a GP reported being bothered by a new health concern associated with their GP consultation in 4.8% of cases, but this was significantly higher among individuals who consulted a GP and a naturopath (10.8%, *p* = 0.01) and those who consulted a GP and an osteopath (12.3%, *p* = 0.002).

### Knowledge and information sharing

The type of information a health professional provided to the participant within a consultation is reported in Table 4. The most common type of information shared with participants during a GP consultation was a verbal explanation (76.3%) followed by an individualised handout (16.8%) and directions on how to access information from another source (11.8%), while 11.6% of these participants reported the GP shared no information with them. Participants who consulted a naturopath reported the naturopath providing a verbal explanation (47.2%), an individualised (36.0%) or a pre-prepared (33.7%) handout, or directions on how to access information from another source (27.0%). Osteopaths were reported to have provided verbal information to 55.1% of the participants who consulted them and individualised handouts to 31.5%.

**Table 4:** Knowledge and information sharing

	Consulted with a GP (*n* = 2002)	Consulted with a naturopath		Consulted with an osteopath (*n* = 89)		Consulted with an acupuncturist	
		Information sharing with naturopath (*n* = 89)	Information sharing with GP (*n* = 74)	Osteopath consultation	GP consultation (*n* = 73)	Acupuncturist consultation	GP consultation (*n* = 74)
Type of information provided by health professional							
*A verbal explanation*	1528 (76.3)	42 (47.2)	42 (56.8)*	49 (55.1)	45 (61.6)*	40 (48.2)	37 (50.0)*
*Individualised handout*	336 (16.8)	32 (36.0)	17 (23.0)	28 (31.5)	23 (31.5)*	23 (27.7)	23 (31.1)*
*Pre-prepared handout*	199 (9.9)	30 (33.7)	14 (18.9)*	20 (22.5)	20 (27.4)	23 (27.7)	15 (20.3)*
*Directions on how to access information from another source*	237 (11.8)	24 (27.0)	19 (25.7)*	20 (22.5)	13 (17.8)	11 (13.3)	14 (18.9)*
*Other*	39 (2.0)	1 (1.1)	1 (1.4)	2 (2.3)	2 (2.7)	0 (0.0)	2 (2.7)
*No information*	233 (11.6)	5 (5.6)	3 (4.1)	2 (2.3)	2 (2.7)*	10 (12.1)	4 (5.4)
Source of information shared with the health professional							
*Personal experience*	1170 (58.4)	42 (47.2)	42 (56.8)	42 (47.2)	40 (54.8)	37 (44.6)	38 (51.4)
*Other health professionals*	410 (20.5)	30 (33.7)	13 (17.6)	29 (32.6)	26 (35.6)*	26 (31.3)	20 (27.0)
*Books*	167 (8.3)	20 (22.5)	10 (13.5)	13 (14.6)	15 (20.6)*	11 (13.3)	14 (18.9)*
*Social media*	116 (5.8)	20 (22.5)	13 (17.6)*	11 (12.4)	12 (16.4)*	8 (9.6)	11 (14.9)*
*Broadcast media*	74 (3.7)	12 (13.5)	6 (8.1)*	11 (12.4)	13 (17.8)*	12 (14.5)	10 (13.5)*
*Friends or family*	139 (6.9)	9 (10.1)	5 (6.8)	9 (10.1)	11 (15.1)*	8 (9.6)	7 (9.5)
*Journal article*	67 (3.4)	7 (7.9)	7 (9.5)*	5 (5.6)	6 (8.2)*	1 (1.2)	6 (8.1)*
*Research organisation*	87 (4.4)	9 (10.1)	4 (5.4)	5 (5.6)	3 (4.1)	5 (6.0)	5 (6.8)
*Government website*	72 (3.6)	4 (4.5)	4 (5.4)	5 (5.6)	6 (8.2)*	1 (1.2)	3 (4.1)
*Other*	17 (0.9)	1 (1.1)	2 (2.7)	0 (0.0)	1 (1.4)	1 (1.2)	1 (1.4)
*None*	553 (27.6)	6 (3.6)	8 (10.8)*	11 (12.4)	11 (15.1)*	10 (12.1)	11 (14.9)*

*p<0.05

Individuals who consulted with a GP and also a naturopath reported a lower rate of receiving a verbal explanation from their GP (56.8%), and a higher rate of their GP giving them a prepared handout (18.9%) or directions on how to access information from another source (25.7%). Participants who consulted a GP and an osteopath also reported a lower rate of receiving a verbal explanation from their GP (61.6%), but a higher rate of being given an individualised handout (31.5%). They also had a much lower rate of receiving no information compared with participants who did not consult an osteopath (2.7%). Participants who consulted an acupuncturist and a GP reported their GP giving them a verbal explanation less frequently than others (50.0%) but a higher rate of all other types of information.

The most common source of information that the participant shared with their GP was personal experience (58.4%) followed by information from other health professionals (20.5%). More than one quarter (27.6%) reported sharing information from none of these sources with their GP. Individuals who consulted a naturopath and a GP reported sharing information from social media (17.6%), a journal article (9.5%), or broadcast media (8.1%) with their GP at a greater rate than other participants. They also reported a lower rate of sharing information from none of these information sources (10.8%). Participants who consulted an osteopath and a GP reported sharing information from other health professionals (35.6%), books (20.6%), social media (16.4%), broadcast media (17.8%), friends or family (15.1%), journal articles (8.2%), and government sources (8.2%) with the GP at a higher rate than participants who did not consult an osteopath. Those who consulted an acupuncturist and a GP reported a statistically higher rate of sharing information with their GP from books (18.9%), social media (14.9%), broadcast media (13.5%) and journal articles (8.1%) at a greater rate than individuals who did not consult an acupuncturist.

### Patient-centred care

The mean score for the patient-centred care scale was between 3.44 and 3.86 for consultations with all health professions studied (see Table 5). The equal highest mean score attributed to consultations with a GP was for the statement ‘I feel seen and heard as an individual’ and ‘my GP is really interested in finding and addressing my health problems’ (mean 3.86), while the lowest score was for ‘my GP receives feedback from my body that guides treatment’ (mean 3.70). The highest mean score attributed to consultations with a naturopath was for ‘my naturopath teaches me ways to relieve symptoms myself’ (mean 3.66) and the lowest was for ‘my naturopath has a full picture of me as an individual’ (mean 3.47). Osteopaths were rated highest for ‘my osteopath asks me for feedback from my body that guides treatment’ (mean 3.80) and lowest for ‘I feel seen and heard as an individual’ (mean 3.55). Acupuncturists’ highest rating was for ‘I know what to expect during consultations and treatment’ (mean 3.69) and lowest for ‘the root causes of my problems are being addressed by my acupuncturist’ (mean 3.38).

**Table 5:** Patient-centred care and empowerment scale (1 = strongly disagree, 5 = strongly agree)

	Consulted with a GP (*n* = 1977) (mean, SD)	Consulted with a naturopath			Consulted with an osteopath (*n* = 87)			Consulted with an acupuncturist		
	Experience of GP consultation	Experience of naturopath consultation (*n* = 89)	Experience of GP consultation (*n* = 72)	P value	Experience of osteopath consultation (*n* = 87)	Experience of GP consultation (*n* = 71)	P value	Experience of acupuncture consultation (*n* = 87)	Experience of GP consultation (*n* = 72)	P value
I feel seen and heard as an individual	3.86 (0.98)	3.56 (1.19)	3.63 (1.04)	0.03	3.55 (1.16)	3.66 (1.09)	0.09	3.56 (1.07)	3.56 (0.99)	0.004
My [health professional] has a full picture of me as an individual	3.83 (0.99)	3.47 (1.05)	3.63 (1.04)	0.05	3.64 (1.02)	3.65 (1.16)	0.2	3.54 (1.07)	3.58 (1.05)	0.02
My [health professional] is really interested in finding and addressing my health problems	3.86 (0.98)	3.55 (1.21)	3.54 (1.15)	0.01	3.59 (1.11)	3.56 (1.11)	0.01	3.61 (1.07)	3.54 (1.04)	0.005
The root causes of my problems are being identified by my [health professional]	3.78 (0.97)	3.48 (0.99)	3.50 (1.05)	0.01	3.66 (0.99)	3.52 (1.18)	0.05	3.38 (1.13)	3.53 (0.98)	0.01
The root causes of my problems are being treated by my [health professional]	3.81 (0.95)	3.51 (1.10)	3.68 (1.09)	0.4	3.85 (0.97)	3.53 (1.18)	0.05	3.55 (1.05)	3.57 (1.10)	0.05
The treatment is individualised for me at each consultation	3.82 (0.94)	3.66 (1.02)	3.88 (0.93)	0.5	3.66 (1.02)	3.61 (1.19)	0.3	3.66 (1.02)	3.60 (0.99)	0.04
My [health professional] receives feedback from my body that guides treatment	3.70 (0.97)	3.53 (1.06)	3.68 (1.01)	0.9	3.65 (1.15)	3.70 (1.09)	0.7	3.67 (0.87)	3.67 (1.01)	0.8
My [health professional] asks me for feedback from my body that guides treatment	3.72 (0.99)	3.63 (0.98)	3.57 (0.95)	0.1	3.80 (1.04)	3.42 (1.09)	0.008	3.44 (1.11)	3.47 (1.01)	0.02
I know what to expect during consultations and treatment	3.96 (0.86)	3.63 (1.09)	3.79 (0.95)	0.2	3.78 (0.97)	3.89 (1.05)	0.9	3.69 (0.99)	3.78 (0.97)	0.09
My [health professional] teaches me ways to relieve symptoms myself	3.69 (0.98)	3.74 (0.99)	3.50 (1.03)	0.1	3.71 (1.02)	3.37 (1.10)	0.008	3.49 (1.04)	3.39 (1.07)	0.009

Compared with participants who did not consult with a naturopath, those who consulted a GP and a naturopath rated their GP lower on feeling seen and heard as an individual (mean 3.63, *p* = 0.04), their GP having a full picture of them as an individual (mean 3.63, *p* = 0.05), their GP being really interested in finding and addressing their health problems (mean 3.54, *p* = 0.01) and the root causes of their problems being identified by their GP (mean 3.50, *p* = 0.01). Individuals who consulted an osteopath rated their GP lower on their GP being really interested in finding and addressing their health problems (mean 3.56, *p* = 0.01), the root cause of their problems being identified by their GP (mean 3.52, *p* = 0.05), the root causes of their problems being treated by their GP (mean 3.53, *p* = 0.05), their GP asking them for feedback from their body that guides treatment (mean 3.42, *p* = 0.008) and their GP teaching them ways to relieve symptoms themself, compared with those who did not consult an osteopath. Participants who consulted an acupuncturist rated their GP lower on each item on the patient-centred care scale except for ‘my GP receives feedback from my body that guides treatment’ and ‘my GP teaches me ways to relieve symptoms myself’, compared to participants who did not consult an acupuncturist.

### Relationship between consultation outcome and information sharing or experience of patient-centred care

As outlined in Table 6, Participants who consulted with a GP and reported receiving an adequate explanation as a result of their consultation were more likely to report their GP provided them with verbal information (OR 6.7; 95% CI 4.7, 9.6), an individualised handout (OR 1.8; 95% CI 1.3, 2.4) or directions on how to access information elsewhere (OR 1.5; 95% CI 1.1, 2.1). No correlation with the provision of a standardised handout or not receiving any information from the GP was identified. Participants were more likely to report receiving an acceptable treatment plan from a GP if they felt the root causes of their health problems were being treated by their GP (OR 1.5; 95% CI 1.3, 1.7) but less likely to report an acceptable treatment plan if they reported that the GP teaches the participant ways to relieve their symptoms themselves (OR 0.8; 95% CI 0.7, 0.9) (see Table 7).

**Table 6:** The likelihood of participants who reported being prescribed an adequate treatment plan as an outcome of their GP consultation also experiencing treatment-relevant characteristics of patient-centred care during their GP consultation (*n* = 1949)

	Odds ratio*	95% CI	p
Information sources			
*Pre-prepared handout*	1.2	0.8, 1.7	0.3
*Directions on how to access information from another source*	1.5	0.1, 2.2	0.02
*Individualised handout*	1.8	1.3, 2.4	< 0.001
*A verbal explanation*	6.7	4.7, 9.6	< 0.001
*No information*	1.1	0.7, 1.7	0.7

*adjusted for confounders: consultations with a naturopath, consultation with an osteopath, consultation with a chiropractor, highest qualification, financial manageability, employment status, relationship status

**Table 7:** The likelihood of the GP providing an information source among participants who reported receiving an adequate explanation as an outcome of their consultation with a GP (*n* = 1949)

	Odds ratio*	95% CI	p
Patient-centred care characteristics			
*The root causes of my problems are being identified by my GP*	1.5	1.3, 1.7	< 0.001
*The treatment is individualised for me at each consultation*	1.1	0.9, 1.3	0.3
*My GP teaches me ways to relieve symptoms myself*	0.8	0.7, 0.9	0.02

*adjusted for confounders: consultations with a naturopath, consultation with an osteopath, consultation with a chiropractor, highest qualification, financial manageability, employment status, relationship status

## Discussion

This study presents novel insights into patient experiences of primary care in Australia, with a specific focus on patient-centred care and knowledge mobilisation across the broad landscape of primary care practice and provision. The crucial role of knowledge mobilisation in addressing contemporary priorities of primary care – such as health literacy and health promotion – underpin the significance of the study findings. One key finding from this analysis is that the vast majority of participants reported receiving verbal health information from their GP and a much smaller proportion received written information. This finding is especially meaningful in the context of increasing efforts in Australia to strengthen the quality of written information provided to patients and the wider community [9]. These efforts have included critical appraisal tools such as the DISCERN instrument, aiming to enhance health literacy by facilitating quality appraisal and higher quality production of written consumer health information [36]. Interestingly with regards to our finding, health literacy research suggests verbal information may offset difficulties in understanding written information [17]. However, such research has commonly focused on structured programs designed specifically to address patient education [37] and may not be directly transferable to interactions occurring as part of routine clinical encounters [38]. These limitations of verbal information may be further compounded when misaligned with a patient’s cultural background or ethnicity [39].

With this context above in mind, it is also noteworthy that patients in our study who also consulted a naturopath, osteopath or acupuncturist reported a lower rate of verbal explanations from their GP compared to other individuals. They also more commonly reported the GP providing a handout or directing them to another information source such as a website. The reason for these findings is unclear and requires further research. However, it could be hypothesized that the lower rate of verbal explanations from a GP influences patients to consult with these other primary care professionals. Alternatively, it is also possible that individuals accessing these other primary care practitioners are not accessing the GP for information and are instead engaging in a more transactional GP encounter to access pharmaceutical prescriptions or medical investigations (e.g., pathology or radiology tests). When considered alongside our finding that participant satisfaction with the explanation provided by a GP was most heavily associated with receiving verbal information, followed by individualised handouts, this focus on provision of verbal information by GPs may in simply reflect patient-centred approaches to communication that cater to patients’ circumstantial preferences. Indeed, survey studies suggest patients prefer verbal information, with many also wanting to receive relevant written handouts [40]. Ultimately, more targeted investigation into the information-sharing behaviours of GP clinical encounters and those encounters involving a broader range of primary care providers is needed.

Over one quarter of study participants who visited a GP did not discuss or share health information with their GP and this rate was lower for individuals who also visited a naturopath, osteopath or acupuncturist. These rates of information-sharing are concerning, particularly with regards to individuals who access multiple forms of health care, as it may suggest that patients are not disclosing the full scope of their health and health care use to primary care professionals, potentially increasing risks associated with unmanaged concomitant use of multiple treatments [28]. The three information sources more commonly shared with a GP among this sub-group of individuals were health information from social media, broadcast media and journal articles. The rate of sharing information from other health professionals to the GP was only greater for survey respondents who also consulted an osteopath. There are a range of reasons individuals may seek health information outside of a clinical encounter including seeking information about medication side effects [15, 41], or attempting to improve their understanding of their own health condition or risks [42]. Patient health information-seeking often reflects patient activation with regards to their health condition [43], however the degree to which a patient is engaged with managing their health does not reduce the need for adequate health literacy to navigate the poor reliability of social and broadcast media information sources they may access as part of their self-management approach. It is unclear what information patients of these other primary care professions are discussing with their GP, and it should be acknowledged that previous research has found health literacy may be greater among some users of these health professions [44]. In contrast to social and broadcast media sources, patients consulting with a naturopath, osteopath or acupuncturist were also found to more commonly discuss information from a journal article with their GP. Overall, our study findings suggest patients may share information from diverse and conflicting sources within a primary care encounter and that this behaviour presents challenges to health professionals seeking to ensure patients are basing their health decisions on accurate information. These challenges are amplified by the fact that primary care practitioners are unlikely to have received formal training in how to engage with patients about misinformation [45] and have limited consultation time to correct misunderstandings and direct patients to correct information [46].

Our study also found participants scored their consultations with a GP as patient-centred, but that these scores were lower among participants who also consulted with at least one other primary care practitioner type included in the study. Patient-centred care is a goal of primary healthcare across the world [47], and has been recently enshrined as critical to effective primary care through the Declaration of Astana [48]. For this reason, the differences in specific patient-centred characteristics of GP consultations reported by participant subgroups in our study warrant closer attention. GP consultations were scored lower across two domains for all three subgroups analysed: *my GP is really interested in finding and addressing my health problems*, and *the root causes of my problems are being identified by my GP*. The belief there is a need for a closer examination of their health may be a motivating factor in seeking other approaches to the health care, such as naturopathy or osteopathy care, especially given both of these health professions and their systems of knowledge emphasise treating underlying health issues [49, 50]. If this need is met through consultation with a primary care practitioner other than their GP, patients may not encourage that dynamic with their GP. Our study does not verify the degree to which other health professionals consulted by these individuals are effective in addressing root causes of their patients’ health problems. It does, however, suggest that some patients desire deeper consideration of their health and if their GP is unable to provide such care, they may seek it elsewhere.

It is also notable that participants in our study who consulted an osteopath or acupuncturist rated their GP lower for the item *My GP teaches me ways to relieve symptoms myself*. This item relates to patient empowerment and the degree to which the practitioner facilitates patient self-care. Self-care is a multifaceted phenomenon that relies in part on patient health literacy, self-awareness, and agency as well as explicit health behaviours such as physical activity and healthy eating [7]. There are also established links between patient empowerment and self-care behaviours [51]. Alongside this growing awareness of the importance of self-care, there is increasing government acknowledgement that primary care practitioners may have a pivotal role in encouraging preventive health behaviours and self-care activities in Australia [5] and internationally [8]. Despite this government recognition, the role of primary care in facilitating patient self-care has received limited research attention [52, 53]. The available research suggests primary care-delivered interventions may improve patient self-care behaviours [53], yet also suggests that primary care practitioners may not be trained in effectively empowering patients to engage in those self-care behaviours [51]. Similarly, while some preliminary research suggests naturopaths [19], osteopaths [22] and acupuncturists [23] often actively encourage patients to improve health behaviours, the degree to which this advice relates to symptom self-management remains unclear. Our study finding of lower GP scores for facilitating self-care may also be because the symptoms experienced by some participants are not easily managed through self-administered techniques. As such, future research should investigate this phenomenon for separate illness populations.

Survey respondents who also visited a naturopath or acupuncturist scored their GP lower for the items pertaining to feeling seen and heard as an individual, and to having a full picture of the patient as an individual. In contrast, participants who consulted an osteopath or acupuncturist rated their GP lower on treating the root causes of their problems and on asking feedback from the patient’s body to guide treatment. As patient-centred care is by its nature tailored to address patients’ unique circumstantial needs, it is possible that some of the differences in items from the patient-centred care scale simply reflect how patient needs are met in different care settings, rather than denoting dissatisfaction. These findings may indicate how patients distinguish between the primary care professions that may best meet their particular needs. Previous research has similarly reported differences in the reasons individuals consult with naturopaths, acupuncturists and manual therapy-based primary care practitioners [27, 54]. For example, patients may consult with an acupuncturist for arthritis [55], while visiting a chiropractor for neck and back pain [56], and a naturopath for female reproductive conditions [57]. This finding reinforces the need for researchers and policymakers to similarly engage with each profession on its own unique characteristics.

### Limitations

Limitations of the study must be considered. Due to the self-report nature of the survey, the study is susceptible to potential recall bias, particularly as participants reported on items from the previous 12 months. It is also important to note that the data related to practitioner practice behaviours is based on patient report and as such may not be an accurate reflection of practitioner behaviour. Furthermore, while the medium of shared information was explored, the quality of information is not known, which should be considered when drawing interpretations from the study findings. The study was also exposed to responder bias as there were some differences in participant characteristics for individuals with missing data associated with some of the analyses, which were adjusted to account for these differences where possible. While the sample was broadly nationally representative, no data were collected on characteristics such as participants’ ethnic or cultural background. This is an important yet complex question in Australia due to the multicultural nature of the population and may add to survey burden, warranting a more focussed exploration through future research to better explore the topic.

Finally, the total number of respondents who identified consulting with a naturopath, osteopath or acupuncturist in our sample did not permit logistic regression analysis to explore the correlation between consultation outcome, information-sharing behaviours and experience of patient-centredness. As such, future research should specifically target users of these health professions to better explore this relationship. Overall, however, the survey included validated instruments used commonly in health services research and a large, representative sample, which afford generalisability of findings to the Australian adult population, increasing their value for researchers, policy makers and health-professionals.

## Conclusion

This study highlights the critical need to better understand the realities of how health literacy and health promotion is addressed in primary care encounters in Australia. The findings suggest primary care practitioners may be relying heavily on verbal information sharing with their patients, and while this method may be preferred by patients the degree to which this method of patient education is effective requires urgent attention. The Australian National Preventive Health Strategy and global calls for patient activation and empowerment for self-care underlines the pivotal role that primary care practitioners continue to have in facilitating healthy, active communities. Public health and health services researchers, policy makers and leaders of primary care professions have a parallel role and responsibility to ensure that primary care practitioners are competent and confident to educate and empower patients in a manner that recognises health literacy needs and the importance of patient-centred care. Attempts to investigate, improve and sustain effective information-sharing and patient-centred approaches to practice in primary care would also do well to pay adequate attention to the inter-professional communication and interface across the complete suite of providers who help make up the primary care landscape.

### Electronic supplementary material

Below is the link to the electronic supplementary material.

Supplementary Material 1

## Data Availability

The datasets analysed during the current study are available from the corresponding author on reasonable request.

## Abbreviations

GP: General practitioner
