# Supplementary material for: Patient experiences of information-sharing and patient-centred care across the broad landscape of primary care practice and provision: a nationally representative survey of Australian adults
Source: BMC Prim Care. 2024 May 4;25:151. doi: 10.1186/s12875-024-02359-8 (PMC11070095; doi:10.1186/s12875-024-02359-8)
Supplement: Supplementary file 1 — Supplementary Material 1 [file 12875_2024_2359_MOESM1_ESM.docx]

CM Use, Health and Disclosure in the Australian Population

Start of Block: Consent to participate

QID48
**About this survey
 Complementary Medicine Use, Health and Disclosure in the Australian Population**
 
WHO IS CONDUCTING THIS RESEARCH?
 My name is Dr Amie Steel and I am a Senior Research Fellow at the Australian Research Centre in Complementary and Integrative Medicine (ARCCIM), University of Technology Sydney (UTS). My colleagues are Distinguished Professor Jon Adams at ARCCIM, UTS, Dr Erica McIntyre, a Research Consultant at the Institute for Sustainable Future, UTS, and Dr Joanna Harnett, a Senior Lecturer at the University of Sydney.
 
WHAT IS THE RESEARCH ABOUT?
 This research is to find out about the patterns of complementary medicine use in the Australian population. It is also designed to explore the patient experience and communication surrounding complementary medicine use in Australia.
 
WHY HAVE I BEEN INVITED?
 You have been invited to participate because you are a member of the Australian population.


WHAT DOES MY PARTICIPATION INVOLVE?
 If you decide to participate, we will invite you to complete an online questionnaire that will take between 15 and 40 minutes of your time, depending on your answers.
 
ARE THERE ANY RISKS/INCONVENIENCE?
 Yes, there is some inconvenience as the survey will take up to 40 minutes to complete. The survey also asks questions about your physical and mental health and your experiences of health care, which may cause some discomfort if you have had distressing experiences in these areas. In the event that you feel distressed, you may wish to:
1. Take a short break for a few minutes
2. Contact one of the free support services listed here: https://www.healthdirect.gov.au/mental-health-helplines
3. Withdraw from the survey at any time without consequences by closing the browser window.
4. If you experience continued feelings of distress after the survey, the services listed in the above link can guide you in the process of seeking ongoing support from a suitable source – you may wish to save the link now for future reference.

 
DO I HAVE TO TAKE PART IN THIS RESEARCH PROJECT?
 Participation in this study is voluntary. It is completely up to you whether or not you decide to take part. If you decide not to participate, or to withdraw from the study, it will not affect your relationship with the researchers or the University of Technology Sydney.
 
WHAT IF I WITHDRAW FROM THIS RESEARCH PROJECT?
 If you wish to withdraw from the survey once it has started, you can do so at any time without having to give a reason, by closing your browser window. However, because your data is anonymous, we are unable to remove your responses if you withdraw.
 
WHAT WILL HAPPEN TO INFORMATION ABOUT ME?
 By ticking the box on the next page and continuing to the survey, you consent to the research team using your questionnaire responses for the research project. All this information will be anonymous. It is anticipated the results of this research will be published and/or presented in a variety of forums. In any publication and/or presentation, information will be provided in such a way that you cannot be identified, as your responses will be anonymous. This survey may be repeated in future to capture changes occurring over time, in which case the data from this project may be used to draw comparisons.
The results of this research may also be shared through open access (public) scientific databases, including internet databases. This will enable other researchers to use the data to investigate other important research questions. Results shared in this way will always be anonymous as you will not be asked to provide any identifying information during the study.
 
WHAT IF I HAVE ANY QUERIES OR CONCERNS?
If you have queries or concerns about the research that you think we can help you with, please feel free to contact us on Amie.Steel@uts.edu.au.
NOTE: This study has been approved in line with the University of Technology Sydney Human Research Ethics Committee [UTS HREC] guidelines. If you have any concerns or complaints about any aspect of the conduct of this research that you wish to raise independently of the research team, please contact the Ethics Secretariat on ph.: +61 2 9514 2478 or email: Research.Ethics@uts.edu.au], and quote the UTS HREC reference number ETH21-6461. Any matter raised will be treated confidentially, investigated and you will be informed of the outcome.
 
**Qualtrics privacy notice**
This survey has been developed by the Australian Research Centre in Complementary and Integrative Medicine, Faculty of Health, at the University of Technology Sydney (UTS). Information is being collected for the purposes outlined above. We invite you to compete this survey. Competing the survey is voluntary and your response to the survey will be anonymous. The information we are collecting will be used as outlined above. Information may also be used and analysed at an aggregate level for quality improvement and planning purposes.
 This survey is provided through Qualtrics. The survey responses will be stored on behalf of UTS by Qualtrics and extracted for use by UTS. Any personal information will not otherwise be disclosed unless you have provided express consent, or where required or permitted by law. Further information on privacy can also be found in the [Qualtrics privacy statement](https://www.qualtrics.com/privacy-statement/).
 If you wish to access or correct information held about you in relation to this survey, contact UTS at the details below. Note that information that is anonymous may not be identified for access or correction. For further information, contact Dr Amie Steel at Amie.Steel@uts.edu.au.

| Page Break |  |
| --- | --- |

| 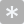 |
| --- |

QID1 **Consent to participate**
 To continue with the survey, read the statements below and indicate your consent by checking the boxes.

- I agree to participate in the research project being conducted by Dr Amie Steel (4)
- I have read the Participant Information Sheet above or someone has read it to me in a language that I understand. (5)
- I understand the purposes, procedures and risks of the research as described in the Participant Information Sheet. (6)
- I have had an opportunity to contact the research team and I am satisfied with the information I have. (7)
- I freely agree to participate in this research project as described and understand that I am free to withdraw at any time without affecting my relationship with the researchers or the University of Technology Sydney. (8)
- I am aware that I can contact Dr Amie Steel (Amie.Steel@uts.edu.au) if I have any concerns about the research. (9)

| Page Break |  |
| --- | --- |

| 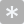 |
| --- |

Q58 What was your age at your last birthday?

________________________________________________________________

| 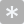 |
| --- |

QID5 What is your residential postcode?

________________________________________________________________

End of Block: Consent to participate

Start of Block: Section 1: Health

QID15 About your health and wellbeing
 The following questions ask you how satisfied you feel, on a scale from 0 to 10. Zero means you feel no satisfaction at all and 10 means you feel completely satisfied.

QID13 Thinking about your own life and personal circumstances...

|  | 0 | 10 |
| --- | --- | --- |

| How satisfied are you with **your life as a whole**? () | 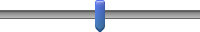 |
| --- | --- |
| How satisfied are you with **your standard of living**? () | 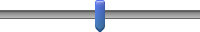 |
| How satisfied are you with **your health**? () | 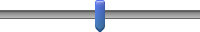 |
| How satisfied are you with **what you are achieving in life**? () | 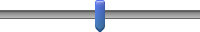 |
| How satisfied are you with **your personal relationships**? () | 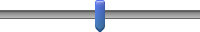 |
| How satisfied are you with **how safe you feel**? () | 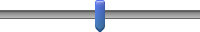 |
| How satisfied are you with **feeling part of your community**? () | 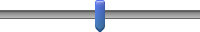 |
| How satisfied are you with **your future security**? () | 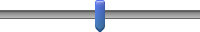 |
| How satisfied are you with **your spirituality and religion**? () | 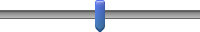 |

| Page Break |  |
| --- | --- |

QID18 In general, would you say your health is:

- Excellent (1)
- Very good (2)
- Good (3)
- Fair (4)
- Poor (5)

| Page Break |  |
| --- | --- |

QID19 For how long (if at all) has your **health limited you** in **each** of the following activities?

|  | Limited for more than 3 months (1) | Limited for less than 3 months (2) | Not limited at all (3) |
| --- | --- | --- | --- |
| The kinds or amounts of **vigorous** activities you do, like lifting heavy objects, running or participating in strenuous sports (1) |  |  |  |
| The kinds or amounts of **moderate** activities you do, like moving a table, carrying groceries or bowling (2) |  |  |  |
| Walking uphill or climbing a few flights of stairs (3) |  |  |  |
| Bending, lifting, or stooping (4) |  |  |  |
| Walking one block (5) |  |  |  |
| Eating, dressing, bathing, or using the toilet (6) |  |  |  |

| Page Break |  |
| --- | --- |

QID20 How much **bodily** pain have you had during the **past 4 weeks**?

- None at all (1)
- A little (2)
- A moderate amount (3)
- A lot (4)
- A great deal (5)

QID21 Does your health **keep you** from working at a job, doing work around the house, or going to school?

- YES, for more than 3 months (1)
- YES, for 3 months or less (2)
- NO (3)

QID22 Have you been unable to do **certain kinds or amounts** of work, housework or schoolwork because of your health?

- YES, for more than 3 months (1)
- YES, for 3 months or less (2)
- NO (3)

| Page Break |  |
| --- | --- |

QID23 For **each** of the following questions, please select the one answer that comes **closest** to the way you have been feeling **during the past month**

|  | All of the time (1) | Most of the time (2) | A good bit of the time (3) | Some of the time (4) | A little of the time (5) | None of the time (6) |
| --- | --- | --- | --- | --- | --- | --- |
| How much of the time, during the past month, has your **health limited your social activities** (like visiting with friends or close relatives)? (1) |  |  |  |  |  |  |
| How much of the time, during the past month, have you been a **very nervous person**? (2) |  |  |  |  |  |  |
| During the past month, how much of the time have you felt **calm and peaceful**? (3) |  |  |  |  |  |  |
| How much of the time, during the past month, have you felt **downhearted and blue**? (4) |  |  |  |  |  |  |
| During the past month, how much of the time have you been a **happy person**? (5) |  |  |  |  |  |  |
| How often, during the past month, have you felt so **down in the dumps that nothing could cheer you up**? (6) |  |  |  |  |  |  |

| Page Break |  |
| --- | --- |

QID24 Please select the answer that **best** describes whether **each** of the following statements is **true** or **false** for you.

|  | Definitely true (1) | Mostly true (2) | Not sure (3) | Mostly false (4) | Definitely false (5) |
| --- | --- | --- | --- | --- | --- |
| I am somewhat ill (1) |  |  |  |  |  |
| I am as healthy as anybody I know (2) |  |  |  |  |  |
| My health is excellent (3) |  |  |  |  |  |
| I have been feeling bad lately (4) |  |  |  |  |  |

| Page Break |  |
| --- | --- |

QID25 In the **last 3 years**, have you been **diagnosed with or treated for** (Mark all that apply):

- type 1 diabetes mellitus (1)
- non-insulin dependent diabetes (2)
- insulin-dependent diabetes (3)
- cancer (benign) (4)
- cancer (malignant) (5)
- heart disease (6)
- hypertension (high blood pressure) (7)
- dislipidaemia (high cholesterol/triglycerides) (8)
- COVID-19 (9)
- chronic fatigue syndrome/myalgic encephalomyelitis (19)
- fibromyalgia (20)
- migraine (59)
- an autoimmune condition (21)
- osteoarthritis (34)
- other musculoskeletal condition (35)
- asthma (36)
- bronchitis (37)
- other respiratory condition (38)
- endometriosis (39)
- polycystic ovary syndrome (PCOS) (40)
- other female reproductive health condition (41)
- benign prostatic hyperplasia (enlarged prostate) (42)
- other male reproductive health condition (43)
- irritable bowel syndrome (IBS) (44)
- inflammatory bowel disease (Crohn's, ulcerative colitis) (45)
- coeliac disease (46)
- gastrooesophageal reflux disease (GERD/GORD) (47)
- chronic constipation (48)
- other gastrointestinal condition (49)
- mood disorder (e.g. depression) (50)
- anxiety disorder (51)
- sleep disorder (52)
- substance use disorder (53)
- schizophrenia or other psychotic disorder (54)
- other mental health condition (55)
- Other long-term health condition (please specify) (56) ________________________________________________
- ⊗None of the above (57)

| Page Break |  |
| --- | --- |

QID14 Are you or your partner currently pregnant?

- Yes (1)
- No (2)
- Unsure (3)

Display This Question:

If Are you or your partner currently pregnant? != Yes

QID16 Are you or your partner currently attempting to become pregnant?

- Yes (1)
- No (2)
- Unsure (3)

QID17 Do you or your partner plan to become pregnant in the next 12 months?

- Yes (1)
- No (2)
- Unsure (3)

End of Block: Section 1: Health

Start of Block: Section 2: Products and Practices

QID42 Did you use any of the following treatments in the **previous 12 months**? Please select all the responses that best apply to you

- prescription-only pharmaceuticals (1)
- over-the-counter pharmaceuticals (2)
- Chinese herbal medicines (3)
- Western herbal medicines (12)
- vitamin/mineral supplements (4)
- yoga practice (5)
- tai chi or qi gong practice (13)
- aromatherapy oils (used externally e.g., applied to the skin or inhaled) (6)
- aromatherapy oils (taken internally) (14)
- homeopathic remedies (7)
- flower essences (8)
- relaxation techniques/meditation/mindfulness practice (9)
- other - please specify: (10) ________________________________________________

Display This Question:

If Did you use any of the following treatments in the previous 12 months? Please select all the resp... = prescription-only pharmaceuticals

Or Did you use any of the following treatments in the previous 12 months? Please select all the resp... = over-the-counter pharmaceuticals

And If

Did you use any of the following treatments in the previous 12 months? Please select all the resp... = Chinese herbal medicines

Or Did you use any of the following treatments in the previous 12 months? Please select all the resp... = Western herbal medicines

Or Did you use any of the following treatments in the previous 12 months? Please select all the resp... = vitamin/mineral supplements

Q60 How often did you take **pharmaceuticals** on the same day as taking **herbal medicines/vitamin/mineral supplements**?

- Never (1)
- Rarely (2)
- Sometimes (3)
- Often (4)
- Always (5)

| Page Break |  |
| --- | --- |

Display This Question:

If Did you use any of the following treatments in the previous 12 months? Please select all the resp... = prescription-only pharmaceuticals

Or Did you use any of the following treatments in the previous 12 months? Please select all the resp... = over-the-counter pharmaceuticals

| 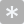 |
| --- |

Q63 On average, how many **pharmaceutical medicines** (prescribed or over-the-counter) did you take **regularly** over the past 12 months?

________________________________________________________________

Display This Question:

If Did you use any of the following treatments in the previous 12 months? Please select all the resp... = Chinese herbal medicines

Or Did you use any of the following treatments in the previous 12 months? Please select all the resp... = Western herbal medicines

Or Did you use any of the following treatments in the previous 12 months? Please select all the resp... = vitamin/mineral supplements

Or Did you use any of the following treatments in the previous 12 months? Please select all the resp... = aromatherapy oils (taken internally)

| 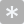 |
| --- |

Q64 On average, how many **complementary medicine products**did you **regularly** take internally over the past 12 months?

 *Complementary medicines are treatments not normally considered part of mainstream healthcare, such as herbal medicine or nutritional supplements*

________________________________________________________________

End of Block: Section 2: Products and Practices

Start of Block: Products and Prescriptions Loop

QID49 You said you used **${lm://Field/1}** in the **previous 12 months**. Who recommended or prescribed them to you? Please select the responses that best apply to you

- General practitioner (GP) (1)
- Specialist doctor (2)
- Hospital doctor (in outpatients or casualty) (9)
- Pharmacist (3)
- Pharmacy/healthfood retail assistant (4)
- Complementary medicine practitioner (e.g., naturopath, acupuncturist, chiropractor, etc) (5)
- Self-selected (6)
- Family member or friend (8)

QID50 And how much do you estimate you have spent on **${lm://Field/1}** in the **previous 12 months?** Write amount below. 
If you did not spend anything, write '**0**'. If you can not recall, write '**unsure**'.

________________________________________________________________

End of Block: Products and Prescriptions Loop

Start of Block: Section 3: Health Service Utilisation

QID30 What was the **frequency of your visits** to the following health professionals in the **previous 12 months**? Please select the responses that best apply to you:

|  |  |
| --- | --- |
| general practitioner (GP) (19) | ▼ None (1) ... More than 6 (5) |
| specialist doctor (20) | ▼ None (1) ... More than 6 (5) |
| hospital doctor (in outpatients or casualty) (21) | ▼ None (1) ... More than 6 (5) |
| pharmacist (22) | ▼ None (1) ... More than 6 (5) |
| counsellor or other mental health worker (23) | ▼ None (1) ... More than 6 (5) |
| community nurse/nurse practitioner (24) | ▼ None (1) ... More than 6 (5) |
| physiotherapist (25) | ▼ None (1) ... More than 6 (5) |
| chiropractor (26) | ▼ None (1) ... More than 6 (5) |
| dietician (27) | ▼ None (1) ... More than 6 (5) |
| osteopath (28) | ▼ None (1) ... More than 6 (5) |
| massage therapist (29) | ▼ None (1) ... More than 6 (5) |
| acupuncturist (30) | ▼ None (1) ... More than 6 (5) |
| naturopath (31) | ▼ None (1) ... More than 6 (5) |
| Western herbalist (32) | ▼ None (1) ... More than 6 (5) |
| traditional Chinese medicine practitioner (33) | ▼ None (1) ... More than 6 (5) |
| homeopath (34) | ▼ None (1) ... More than 6 (5) |
| yoga teacher (36) | ▼ None (1) ... More than 6 (5) |
| Other - please specify (or select 'none') (37) | ▼ None (1) ... More than 6 (5) |

End of Block: Section 3: Health Service Utilisation

Start of Block: Condition-Profession Loop

Carry Forward Unselected Choices from "What was the frequency of your visits to the following health professionals in the previous 12 months? Please select the responses that best apply to you:"

| 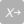 |
| --- |

QID35 Which, if any, of the following health care providers did you visit in the **previous 12 months** specifically **for your ${lm://Field/1}**?

- general practitioner (GP) (1)
- specialist doctor (2)
- hospital doctor (in outpatients or casualty) (3)
- pharmacist (4)
- counsellor or other mental health worker (5)
- community nurse/nurse practitioner (6)
- physiotherapist (7)
- chiropractor (8)
- dietician (9)
- osteopath (10)
- massage therapist (11)
- acupuncturist (12)
- naturopath (13)
- Western herbalist (14)
- traditional Chinese medicine practitioner (15)
- homeopath (16)
- yoga teacher (17)
- Other - please specify (or select 'none') (18) ________________________________________________

End of Block: Condition-Profession Loop

Start of Block: Health Service Ute Loop

QID34 What was the **reason** for visiting a**${lm://Field/1}** in the **previous 12 months**? Please select the responses that best apply to you.

- For an acute illness/condition, one that lasted less than 1 month (1)
- To treat a long-term health condition (one that lasted more than 1 month), or its symptoms (2)
- To improve wellbeing (3)
- Other - please specify (4) ________________________________________________

| 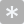 |
| --- |

QID37 What was the estimated cost to you **per ${lm://Field/1} visit** ($) - consultation fees only

________________________________________________________________

| Page Break |  |
| --- | --- |

QID41 How did you attend your consultation/s with a **${lm://Field/1} in the previous 12 months**? (select all that apply)

- In person (1)
- Telehealth (by phone or computer) (2)

QID27 What was the **outcome** of your visit with your **${lm://Field/1}**? (select all that apply)

- I was provided an adequate explanation of my health complaint (1)
- I was provided a formal diagnosis of my health condition (2)
- I was prescribed an acceptable treatment plan to manage my health complaint (3)
- I am still bothered by the same health concern (4)
- I am bothered by a new health concern (5)
- Other - please specify: (6) ________________________________________________

| Page Break |  |
| --- | --- |

QID60 During your visit, did the **${lm://Field/1}** provide you with any of the following types of information? (Select all that apply)

- A verbal explanation (1)
- An individualised handout they gave to you during your visit (2)
- A pre-prepared handout that they gave to you during your visit (7)
- Directions on how to access information from another source (e.g. website or book) after your visit (8)
- Other - Please describe (9) ________________________________________________
- ⊗They did not provide me with any information (10)

| Page Break |  |
| --- | --- |

QID62 During your visit with the **${lm://Field/1}**, did you share any of the following types of knowledge or information with them? (select all that apply)

- Knowledge based on your personal experience of living with your health condition (1)
- Information you acquired when visiting with another health professional (2)
- Information from books you have read (10)
- Information you found through social media (11)
- Information you found through broadcast media (e.g. radio, television) (12)
- Information you found through friends and family (13)
- Information you read in a journal article (14)
- Information you found through a research organisation (15)
- Information you found through a government website (16)
- Other - please describe (17) ________________________________________________
- ⊗I did not share any knowledge or information with them (18)

| Page Break |  |
| --- | --- |

QID64 During your visit with a **${lm://Field/1}**, please indicate whether you **accessed care for**: (select all that apply)

- COVID symptoms (after diagnosis) (1)
- COVID symptoms (before diagnosis) (2)
- Prevention of COVID (3)
- ⊗None of the above (7)

QID65 During your visit with a **${lm://Field/1}**, please indicate whether you **discussed**: (select all that apply)

- COVID-related information (1)
- COVID vaccination information (2)
- ⊗None of the above (7)

| Page Break |  |
| --- | --- |

QID31 When thinking about your **most recent visit**with your **${lm://Field/1}**, how much do you agree or disagree with the following statements?

|  | Strongly disagree (1) | Disagree (2) | Neutral (3) | Agree (4) | Strongly agree (5) |
| --- | --- | --- | --- | --- | --- |
| I feel seen and heard as a unique individual by my ${lm://Field/1} (1) |  |  |  |  |  |
| My ${lm://Field/1} has a full picture of me as a unique individual (2) |  |  |  |  |  |
| My ${lm://Field/1} is really interested in finding and addressing my health problems (3) |  |  |  |  |  |
| The root causes of my problems are **identified** by my ${lm://Field/1} (4) |  |  |  |  |  |
| The root causes of my problems are being **treated** by my ${lm://Field/1} (5) |  |  |  |  |  |
| The treatment is individualised for me at each consultation (6) |  |  |  |  |  |
| My ${lm://Field/1} **receives** feedback from my body that guides treatment (7) |  |  |  |  |  |
| My${lm://Field/1} **asks me** for feedback from my body that guides treatment (8) |  |  |  |  |  |
| I know what to expect during consultations and treatment (9) |  |  |  |  |  |
| My ${lm://Field/1} teaches me ways to relieve symptoms myself (10) |  |  |  |  |  |

| Page Break |  |
| --- | --- |

Display This Question:

If Loop 19: general practitioner (GP), Current Loop

Or Loop 20: specialist doctor, Current Loop

Or Loop 21: hospital doctor (in outpatients... Current Loop

Or Loop 22: pharmacist, Current Loop

And If

Did you use any of the following treatments in the previous 12 months? Please select all the resp... = Chinese herbal medicines

Or Did you use any of the following treatments in the previous 12 months? Please select all the resp... = Western herbal medicines

Or Did you use any of the following treatments in the previous 12 months? Please select all the resp... = vitamin/mineral supplements

QID39
The following question relates to the disclosure of information about your use of health treatments to health professionals providing your health care in the **previous 12 months.**

 *Complementary medicines are treatments not normally considered part of mainstream healthcare, such as herbal medicine, nutritional supplements or homeopathy*
 Please select the response that best reflects your experience with your **${lm://Field/1}**...

- I told them about ALL complementary medicines I was using (1)
- I only told them about SOME of my complementary medicine use (2)
- I DID NOT tell them about my complementary medicine use (3)

| Page Break |  |
| --- | --- |

Display This Question:

If Loop current: The following question relates to the disclosure of information about your use of health treatmen... = I only told them about SOME of my complementary medicine use

Or Loop current: The following question relates to the disclosure of information about your use of health treatmen... = I DID NOT tell them about my complementary medicine use

QID40
The following questions relate to your interactions with a medical doctor in the **last 12 months**. Please indicate your level of agreement with the following statements
 
I **did not disclose** my complementary medicine use to my **${lm://Field/1}** because...

|  | Strongly Agree (1) | Agree (2) | Neutral (3) | Disagree (4) | Strongly Disagree (5) |
| --- | --- | --- | --- | --- | --- |
| They did not ask me about my complementary medicine use (1) |  |  |  |  |  |
| I did not think they would understand my choice (2) |  |  |  |  |  |
| I was worried they would judge me (3) |  |  |  |  |  |
| Complementary medicines are safe (4) |  |  |  |  |  |
| They did not need to know (5) |  |  |  |  |  |
| There was not enough time in the consultation (6) |  |  |  |  |  |
| I felt uncomfortable discussing it with them (7) |  |  |  |  |  |
| I was worried they wouldn't support my treatment decisions (8) |  |  |  |  |  |
| I did not think they would know anything about complementary medicine (9) |  |  |  |  |  |
| It is none of their business (10) |  |  |  |  |  |
| I was worried they would try to discourage my use of complementary medicine (11) |  |  |  |  |  |
| They do not approve of my use of complementary medicine (12) |  |  |  |  |  |
| I was worried they would respond negatively (13) |  |  |  |  |  |
| I previously had a negative experience when I disclosed complementary medicine use (14) |  |  |  |  |  |

| Page Break |  |
| --- | --- |

Display This Question:

If Loop current: The following question relates to the disclosure of information about your use of health treatmen... = I told them about ALL complementary medicines I was using

Or Loop current: The following question relates to the disclosure of information about your use of health treatmen... = I only told them about SOME of my complementary medicine use

QID36
The following questions relate to your interactions with a medical doctor in the **last 12 months**. Please indicate your level of agreement with the following statements
 
I **disclosed** my complementary medicine use to my **${lm://Field/1}** because...

|  | Strongly Agree (1) | Agree (2) | Neutral (3) | Disagree (4) | Strongly Disagree (5) |
| --- | --- | --- | --- | --- | --- |
| I wanted them to fully understand my health status (1) |  |  |  |  |  |
| I was concerned about drug interactions with the complementary medicine I was using (2) |  |  |  |  |  |
| I thought they might know something useful about complementary medicine (3) |  |  |  |  |  |
| The asked me about my use of complementary medicine (4) |  |  |  |  |  |
| I have a good relationship with them (5) |  |  |  |  |  |
| I felt comfortable discussing complementary medicine with them (6) |  |  |  |  |  |
| I knew they would be willing to discuss my complementary medicine use (7) |  |  |  |  |  |
| I wanted their approval of my complementary medicine use (8) |  |  |  |  |  |
| I knew they would understand about my complementary medicine use (9) |  |  |  |  |  |
| They have a good attitude towards complementary medicine (10) |  |  |  |  |  |
| They are open-minded (11) |  |  |  |  |  |
| I thought they could help with my treatment decisions (12) |  |  |  |  |  |
| They support my use of complementary medicine (13) |  |  |  |  |  |
| They understand my treatment goals (14) |  |  |  |  |  |
| They have my best interests at heart (15) |  |  |  |  |  |
| I wanted their advice about complementary medicines (16) |  |  |  |  |  |

| Page Break |  |
| --- | --- |

Display This Question:

If Loop 26: chiropractor, Current Loop

Or Loop 28: osteopath, Current Loop

Or Loop 29: massage therapist, Current Loop

Or Loop 30: acupuncturist, Current Loop

Or Loop 31: naturopath, Current Loop

Or Loop 32: Western herbalist, Current Loop

Or Loop 33: traditional Chinese medicine pr... Current Loop

Or Loop 34: homeopath, Current Loop

And If

Did you use any of the following treatments in the previous 12 months? Please select all the resp... = prescription-only pharmaceuticals

Or Did you use any of the following treatments in the previous 12 months? Please select all the resp... = over-the-counter pharmaceuticals

QID44
The following questions relate to the **disclosure** of information about your use of **conventional medicines** to **complementary medicine practitioners** providing you health care.

 *Conventional medicines are treatments that are considered part of mainstream healthcare, such as pharmaceutical drugs.*
 
Please select the response that best reflects your experience with your **${lm://Field/1}** in the **previous 12 months**...

- I told them about ALL conventional medicines I was using (1)
- I only told them about SOME of my conventional medicine use (2)
- I DID NOT tell them about my conventional medicine use (3)

| Page Break |  |
| --- | --- |

Display This Question:

If Loop current: The following questions relate to the disclosure of information about your use of conventional me... = I only told them about SOME of my conventional medicine use

Or Loop current: The following questions relate to the disclosure of information about your use of conventional me... = I DID NOT tell them about my conventional medicine use

QID52
The following questions relate to your interactions with complementary medicine practitioners (e.g. naturopath, herbalist, homeopath) in the **last 12 months**. Please indicate your level of agreement with the following statements
 
I **did not disclose** my conventional medicine use to my **${lm://Field/1}** because...

|  | Strongly Agree (1) | Agree (2) | Neutral (3) | Disagree (4) | Strongly Disagree (5) |
| --- | --- | --- | --- | --- | --- |
| They did not ask me about my conventional medicine use (1) |  |  |  |  |  |
| I did not think it was important (2) |  |  |  |  |  |
| I did not think they would understand my choice (3) |  |  |  |  |  |
| I was worried they would judge me (4) |  |  |  |  |  |
| They did not need to know (5) |  |  |  |  |  |
| There was not enough time in the consultation (6) |  |  |  |  |  |
| I felt uncomfortable discussing it with them (7) |  |  |  |  |  |
| I was worried they wouldn't support my treatment decisions (8) |  |  |  |  |  |
| I did not think they would know anything about conventional medicines (9) |  |  |  |  |  |
| I forgot to mention it (10) |  |  |  |  |  |
| It is none of their business (11) |  |  |  |  |  |
| I was worried they would try to discourage my use of conventional medicines (12) |  |  |  |  |  |
| They do not approve of my use of conventional medicines (13) |  |  |  |  |  |
| I was worried they would respond negatively (14) |  |  |  |  |  |
| I do not use conventional medicines regularly enough (15) |  |  |  |  |  |
| I previously had a negative experience when I disclosed using conventional medicine (16) |  |  |  |  |  |

| Page Break |  |
| --- | --- |

Display This Question:

If Loop current: The following questions relate to the disclosure of information about your use of conventional me... = I told them about ALL conventional medicines I was using

Or Loop current: The following questions relate to the disclosure of information about your use of conventional me... = I only told them about SOME of my conventional medicine use

QID45
The following questions relate to your interactions with complementary medicine practitioners (e.g. naturopath, herbalist, homeopath) in the **last 12 months**. Please indicate your level of agreement with the following statements
 
I **disclosed** my conventional medicine use to my **${lm://Field/1}** because...

|  | Strongly Agree (1) | Agree (2) | Neutral (3) | Disagree (4) | Strongly Disagree (5) |
| --- | --- | --- | --- | --- | --- |
| I wanted them to fully understand my health status (1) |  |  |  |  |  |
| I was concerned about interactions with the conventional medicine I was using (2) |  |  |  |  |  |
| I thought they might know something about conventional medicines (3) |  |  |  |  |  |
| They asked me about my use of conventional medicines (4) |  |  |  |  |  |
| I have a good relationship with them (5) |  |  |  |  |  |
| I felt comfortable discussing conventional medicines with them (6) |  |  |  |  |  |
| I knew they would be willing to discuss my conventional medicine use (7) |  |  |  |  |  |
| I wanted their approval of my conventional medicine use (8) |  |  |  |  |  |
| I knew they would understand about my conventional medicine use (9) |  |  |  |  |  |
| They have a good attitude towards conventional medicine (10) |  |  |  |  |  |
| They are open-minded (11) |  |  |  |  |  |
| I thought they could help with my treatment decisions (12) |  |  |  |  |  |
| They support my use of conventional medicines (13) |  |  |  |  |  |
| They understand my treatment goals (14) |  |  |  |  |  |
| They have my best interests at heart (15) |  |  |  |  |  |
| I was concerned about side-effects of conventional medicines (16) |  |  |  |  |  |
| I wanted their advice about conventional medicines (17) |  |  |  |  |  |

End of Block: Health Service Ute Loop

Start of Block: Ethics Risk/Harm reminder for potential distress

Ethics
We appreciate the responses you have provided so far regarding your health and experiences of health care.
If these questions have caused you any discomfort or distress, remember that you can access free support services at this link:
 <https://www.healthdirect.gov.au/mental-health-helplines>
 You are almost at the end of the survey.

End of Block: Ethics Risk/Harm reminder for potential distress

Start of Block: Section 4: Demographics

QID2 About You
 Which gender do you identify with?

- Female (1)
- Male (2)
- Non-binary (3)
- Prefer to self-describe/other (please specify): (4) ________________________________________________

QID4 What is your age range?

- 18-24 (1)
- 25-34 (2)
- 35-44 (3)
- 45-54 (4)
- 55-64 (5)
- 65 and over (6)

| Page Break |  |
| --- | --- |

QID6 How do you manage financially at the moment?

- It is impossible (1)
- It is difficult all of the time (2)
- It is difficult some of the time (3)
- It is not too bad (4)
- It is easy (5)

QID7 What is the highest qualification you have completed?

- No formal qualifications (1)
- Year 10 or equivalent (2)
- Year 12 or equivalent (3)
- Trade/apprenticeship (4)
- Certificate/diploma (5)
- University degree (6)
- Higher university degree (e.g. Masters, PhD) (7)

| Page Break |  |
| --- | --- |

QID8 Do you currently have private health insurance?

- Yes (1)
- No (2)

QID9 Do you currently have a Health Care Card?

- Yes (1)
- No (2)

QID10 What best describes your current employment status?

- Full time work (35 or more hours per week) (1)
- Part time work (less than 35 hours per week) (2)
- Casual/temp work (irregular hours) (3)
- Looking for work (4)
- Not in the paid workforce (5)

| Page Break |  |
| --- | --- |

QID11 What is your present relationship status?

- Never married (1)
- Married (opposite sex) (2)
- Married (same sex) (3)
- De facto (opposite sex) (4)
- De facto (same sex) (5)
- Separated (6)
- Divorced (7)
- Widowed (8)

| Page Break |  |
| --- | --- |

Display This Question:

If Do you currently have private health insurance? = Yes

QID12 If you currently have private health insurance for ancillary services, please indicate which services are covered:

- ⊗I do not have private health insurance for ancillary services (1)
- Physiotherapy (2)
- Psychology services (3)
- Chiropractic (4)
- Osteopathy (5)
- Acupuncture (6)
- Chinese medicine/herbs (7)
- Remedial massage/massage therapy (8)
- Nutrition/dietetics (9)
- ⊗Not sure (10)

| Page Break |  |
| --- | --- |

End of Block: Section 4: Demographics

Start of Block: Block 9

End We appreciate the responses you have provided today.
 If any questions have caused you discomfort or distress, remember that you can access free support services at this link:
 [https://www.healthdirect.gov.au/mental-health-helplines](http://www.healthdirect.gov.au/mental-health-helplines)

End of Block: Block 9
